# Phenomenology, clinical aspects and therapeutic implications of delusional memories in Delusional Disorders: A Systematic Review

**DOI:** 10.1192/j.eurpsy.2023.809

**Published:** 2023-07-19

**Authors:** A. González- Rodríguez, J. A. Monreal, M. Solmi, M. Balestrieri, M. Fornaro, A.-L. Panfil, F. Duval, M. V. Seeman

**Affiliations:** 1 Mental Health, Mutua Terrassa University Hospital. University of Barcelona (UB). CIBERSAM. DEDIWoG; 2 Mental Health, Mutua Terrassa University Hospital. University of Barcelona (UB). CIBERSAM. Inst. Neurosc. UAB. DEDIWoG, Terrassa, Spain; 3 Psychiatry, University of Otawa. DEDIWoG, Otawa, Canada; 4 Psychiatry, University of Udine. DEDIWoG, Udine; 5 Psychiatry, Federico II University of Naples. DEDIWoG, Naples, Italy; 6 Liaison Psychiatry, Pius Brinzeu County Emergency Hospital. DEDIWoG, Timisoara, Romania; 7 Pôle 8/9-APF2R, Centre Hospitalier. DEDIWoG, Rouffach, France; 8 Psychiatry, University of Toronto. DEDIWoG, Toronto, Canada

## Abstract

**Introduction:**

Delusional memories or retrospective delusions have been extensively reported in subjects during or after intensive care stays. In major psychoses, authors have classically observed delusional memories impacting the prognosis and mental well-being.

**Objectives:**

Our aim was to review the phenomenology, psychological/biological factors contributing to delusional memories in delusional disorder (DD), and potential treatment strategies.

**Methods:**

Systematic review using PubMed, Scopus, SciELO and Web of Science electronic databases (inception-September 2022). Search terms: (“delusional memories” OR “retrospective delusions”) AND (“Schizophrenia, Paranoid”) [MeSH]. Studies were included if they reported psychopathology, clinical characteristics or treatment strategies of “delusional memories” in DD. Team members: AGR, JAM, MS, MB, MF, ACP, FD, MVS.

**Results:**

A total of 786 records were retrieved, including six studies. Psychogenesis:A novel cognitive neuropsychological research model (based on hypnosis) in erotomania delusions suggest a potential recall and reinterpretation of delusions beliefs in highly hypnotizable subjects. Biological basis: Frontal lobe (or executive) dysfunction does not seem to contribute to delusional memories in De Clérambault syndrome (erotomania). Phenomenology: 1)General knowledge was essentially intact, while the perceptual characteristics of delusional memories were stronger than real memories. 2)Correlations were found between delusional ideation, positive dimension of schizotypy (r=0.18), and false memories (r=0.27). 3)Jumping-to-conclusions and liberal acceptance bias influence delusional memories. Treatment:Efficacy of 1)Cognitive Behavioural Therapy (CBT) (significant reduction delusions), and 2)Metacognitive control over false memories.

**Conclusions:**

This is the first review exploring the genesis and management of delusional memories in DD. Memory deficits/executive dysfunctions do not seem to be the only cause of this phenomenon.

**Disclosure of Interest:**

None Declared

